# Deconfounding Phenology in SPAD-Based Rice Nitrogen Diagnosis Using Physiological Time and Canopy-Stratified Measurements

**DOI:** 10.3390/plants15040591

**Published:** 2026-02-13

**Authors:** Chengyingying Qin, Haitao Xiang, Qiaoyi Huang, Yuan Wang

**Affiliations:** 1 State Key Laboratory of Soil and Sustainable Agriculture, Changshu National Agro-Ecosystem Observation and Research Station, Institute of Soil Science, Chinese Academy of Sciences, Nanjing 210008, China; qinchengyingying@issas.ac.cn (C.Q.); htxiang@issas.ac.cn (H.X.); 2 University of Chinese Academy of Sciences, Beijing 100049, China; 3 University of Chinese Academy of Sciences, Nanjing 211135, China; 4 Institute of Agricultural Resources and Environment, Guangdong Academy of Agricultural Sciences, Guangzhou 510640, China; huangqiaoyi@gdaas.cn

**Keywords:** rice, SPAD, growing degree days, physiological time, nitrogen nutrition index, critical nitrogen dilution, machine learning

## Abstract

Phenology can confound rice nitrogen diagnosis based on SPAD readings because leaf greenness and nitrogen concentration change nonlinearly with development. We tested whether physiological time, expressed as growing degree days (GDD), can reduce this developmental bias and improve the portability of SPAD-based diagnosis. We analyzed 1141 observations from 20 independent field experiments across five sites, spanning japonica, indica, and hybrid cultivars and nitrogen fertilizer treatments (0–300 kg N ha^−1^). SPAD was measured on up to five leaf-from-top positions (LFT1–LFT5) and used to predict leaf nitrogen concentration (LNC), plant nitrogen concentration (PNC), and nitrogen nutrition index (NNI). Across group-wise cross-validation by experiment, adding GDD to SPAD consistently improved cross-environment accuracy (mean R^2^ up to 0.75 for LNC and 0.79 for PNC) and markedly weakened residual trends along GDD. Multiplicative SPAD×GDD degraded performance, while explicit interaction terms provided little gain over a simple additive SPAD + GDD form. Interpretable analyses further showed that diagnostic information is concentrated in mid-canopy leaves and shifts with physiological time. Combining GDD with a two-leaf SPAD protocol retained most accuracy for concentration targets, supporting a time-aligned and field-practical approach for robust nitrogen diagnosis.

## 1. Introduction

Nitrogen (N) is the primary macronutrient limiting rice productivity, as it underpins chlorophyll formation, photosynthetic capacity, and the biomass production that ultimately supports grain filling [1,2]. Yet the dominant historical approach—high input to secure high yield—has become increasingly difficult to justify as agronomic returns plateau while environmental externalities intensify [3,4]. Across intensive cereal systems, a substantial fraction of applied N is not recovered by crops, instead contributing to reactive N losses, water eutrophication risks, soil acidification, and enhanced greenhouse-gas emissions [5,6]. A practical pathway out of this dilemma is precision nitrogen management, which aims to synchronize N supply with crop demand in both time and space [7,8]. The central prerequisite for doing so is an in-season, field-deployable diagnosis of crop N status that is sufficiently robust to genotypic and environmental variability [9,10].

Conventional wet-chemistry methods (e.g., Kjeldahl or combustion-based assays) provide accurate N quantification but are destructive, labor-intensive, and too slow to support tactical topdressing decisions within narrow agronomic windows. Optical surrogates therefore became a cornerstone of operational diagnostics [11]. Among them, the chlorophyll meter (Soil–Plant Analysis Development, SPAD) has been widely adopted because it is rapid, portable, and non-destructive, and because leaf greenness is physiologically linked to N allocation within chloroplasts [12,13,14,15]. In practice, SPAD-based decision rules have progressed from fixed thresholds toward more transferable approaches such as reference-based and sufficiency-style comparisons, and are increasingly integrated with broader sensing and decision-support workflows. Nevertheless, several persistent challenges limit robust deployment, including limited transferability across cultivars and environments, sensitivity to leaf structural/optical properties, and confounding by plant developmental processes that can shift the SPAD–N relationship over time [13,16,17].

However, SPAD is not a pure proxy for N supply, as it can conflate nutritional status with developmental progression [13]. As rice develops, the greenness–N relationship shifts due to growth-linked N dilution (critical N dilution theory and rice-specific implementations) [18,19,20], age-dependent changes in leaf structure that affect optical transmittance [21,22,23], and senescence and N remobilization during reproductive development that generate physiologically ‘normal’ chlorosis patterns [24,25,26]. Together, these processes induce phenology-driven drift, such that a single SPAD-based diagnostic rule can systematically change across the season even under comparable N supply [27].

A second practical complication is that SPAD signals are vertically heterogeneous within the canopy. Leaf position modulates both SPAD values and their diagnostic meaning because canopy stratification reflects coordinated N allocation under light gradients and age-dependent turnover. Empirically, SPAD distributions vary with N rate and leaf age [12,28], and the ‘optimal’ measurement leaf is not universal [13,29]. Because this vertical structure can be agronomically informative [30,31], robust SPAD-based diagnosis must address two coupled deployment questions: how to account for time (phenology) and which leaf positions to measure, potentially in a target-dependent manner.

Here we argue that phenological bias is best handled as a physiological time-alignment problem. Thermal time, quantified as growing degree days (GDD) [32], provides a biologically grounded index of developmental progress [9,33]. By placing SPAD observations on a common physiological timeline, GDD enables principled comparisons across dates, genotypes, and environments [10,34], and motivates an additive SPAD + GDD structure in which SPAD captures instantaneous greenness while GDD accounts for predictable time-structured shifts associated with dilution, aging, and remobilization. Accordingly, we ask whether GDD can deconfound phenology-driven bias in multi-leaf SPAD signals to diagnose rice N status robustly across genotypes and environments, while clarifying which leaf positions matter, when, and for which targets (LNC, PNC, and NNI). Specifically, we aim to (i) identify a model form that corrects systematic phenological drift; (ii) quantify how the relative diagnostic value of leaf positions changes along physiological time and across targets; and (iii) translate these insights into a simplified, field-feasible measurement protocol with minimal sampling burden.

## 2. Results

### 2.1. Dataset Variability Supports Stringent Testing of Nitrogen Diagnostics

Across 20 independent field experiments, the dataset spans a wide N gradient, with substantial variability in both leaf nitrogen concentration (LNC; SD = 0.82) and plant nitrogen concentration (PNC; SD = 0.76), supporting robust model evaluation beyond site-specific fitting. Raw SPAD showed a mild decline in mean values from upper to lower leaves (e.g., LFT2 mean 42.43 vs. LFT5 mean 39.17), while dispersion increased toward older leaves, consistent with position-dependent senescence and increasing signal heterogeneity later in the season (Table 1).

### 2.2. Additive SPAD + GDD Alignment Reduces Phenology-Related Bias and Improves Cross-Environment Accuracy

**Figure 1:**
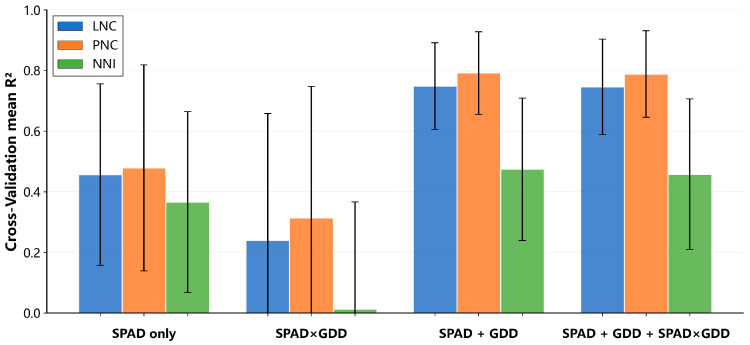
Cross-validated mean R^2^ for predicting LNC, PNC, and NNI under four SPAD–GDD feature constructions. Bars summarize the mean R^2^ aggregated across PLS, SVR, RF, and XGBoost (10-fold GroupKFold by experiment). Error bars indicate variability (Standard Deviation).

Using group-aware evaluation (10-fold GroupKFold by experiment, N = 1141), the additive SPAD + GDD formulation consistently outperformed SPAD-only for all targets (Figure 1; Table 2), with the largest gains for concentration traits (mean R^2^ up to 0.75 for LNC and 0.79 for PNC). Improvements for NNI were smaller but consistent. In contrast, the multiplicative SPAD×GDD formulation degraded performance, and adding explicit interaction terms provided little additional benefit over the simple additive SPAD + GDD form (Figure 1; Table 2).

**Figure 2:**
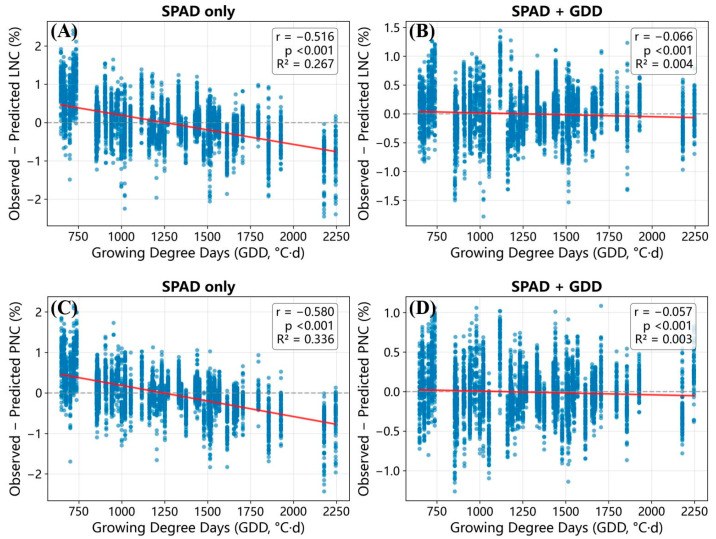
Residual diagnostics along physiological time (GDD) for SPAD-only versus SPAD + GDD models. Residuals are computed as Observed−Predicted for (**A**,**B**) LNC and (**C**,**D**) PNC. Each target contains two panels: SPAD-only (left) and SPAD + GDD (right). The solid red line is the fitted linear trend against GDD; the horizontal dashed line marks zero residual.

Residual diagnostics along the physiological-time axis provided direct evidence that adding GDD reduces phenology-related systematic error (Figure 2). Under SPAD-only, out-of-fold residuals showed a strong negative association with GDD, indicating stage-dependent directional error. After adding GDD, these residual–GDD relationships largely collapsed, consistent with reduced sensitivity of prediction error to developmental progression (Figure 2).

**Figure 3:**
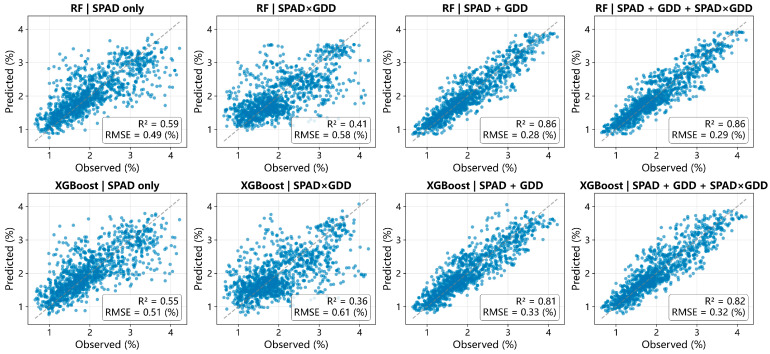
Observed PNC versus predicted PNC for RF and XGBoost under four feature constructions. Panels show 1:1 scatterplots for RF (top row) and XGBoost (bottom row), with columns corresponding to feature constructions (SPAD only, SPAD×GDD, SPAD + GDD, SPAD + GDD + SPAD×GDD). Each panel includes a 1:1 dashed line and annotated R^2^ and RMSE.

Algorithm-specific 1:1 plots further indicate that the performance gains from SPAD + GDD were not restricted to a single learning method (Figure 3). Both RF and XGBoost showed improved agreement between observed and predicted values under additive formulations, while SPAD×GDD produced more dispersed predictions. Across all model–feature combinations, the best-performing configurations were obtained with tree ensembles under additive features (Table 2), supporting the robustness of the formulation-level comparison.

### 2.3. Target-Dependent Reliance on Physiological Time (GDD) Versus Canopy SPAD Signals

**Figure 4:**
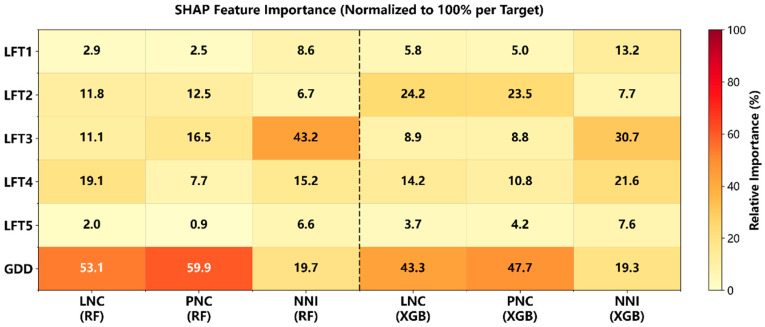
Target-wise feature attribution heatmap for RF and XGBoost models. Heatmap of normalized feature importance (%) for predicting LNC, PNC, and NNI using GDD and SPAD measured on the 1st–5th fully expanded leaves from the top (LFT1–LFT5). Importance values were derived from 10-fold cross-validation and normalized to sum to 100% within each target–model combination. LNC and PNC are in %, NNI is dimensionless. Importance values represent model-based attribution and do not imply causality.

Feature attribution indicated that the relative contribution of physiological time (GDD) versus canopy SPAD is strongly target dependent (Figure 4). For concentration traits (LNC and PNC), GDD accounted for a large share of the predictive signal (≈43–60% across RF and XGBoost), whereas for NNI the contribution of GDD was much smaller (≈19%), indicating stronger reliance on canopy SPAD structure. Across targets and learners, the SPAD signal was concentrated in the mid-canopy leaves (LFT2–LFT4) (Figure 4). This mid-canopy dominance was most pronounced for NNI, where LFT3 provided the largest contribution (43.2% in RF; 30.7% in XGBoost) and LFT4 contributed substantially (15.2% in RF; 21.6% in XGBoost). For the concentration targets, mid-canopy contributions remained prominent but with target- and learner-specific emphasis. For LNC, the dominant SPAD contributor was LFT4 under RF (19.1%), whereas XGBoost emphasized LFT2 (24.2%) with additional weight on LFT4 (14.2%). For PNC, RF assigned its largest SPAD weight to LFT3 (16.5%) alongside LFT2 (12.5%), while XGBoost again highlighted LFT2 (23.5%) as the leading SPAD feature. In contrast, LFT1 remained low across all targets (≤13.2%), and LFT5 was consistently minor (≤7.6%), consistent with reduced incremental diagnostic value from the newest leaf and limited availability of the lowest leaf later in development.

### 2.4. Dynamic Shifts in Mid-Canopy Leaf Feature Importance Along Physiological Time

**Figure 5:**
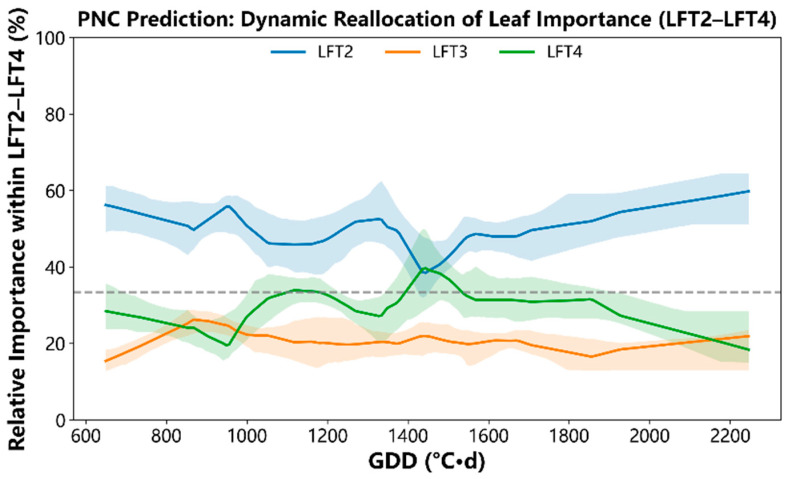
Dynamic reallocation of mid-canopy leaf feature importance along physiological time. Curves show the LOWESS-smoothed relative feature importance (%) of SPAD measured at LFT2–LFT4 as a function of GDD. For each observation, absolute attributions were normalized within LFT2–LFT4 to sum to 100%, highlighting redistribution among the key leaf positions. Shaded regions denote bootstrap confidence bands. The vertical dashed line marks the crossover where LFT2 and LFT3 contribute equally (1066 °C·d), and the translucent band indicates its 95% bootstrap confidence interval (1042–1138 °C·d). The horizontal dashed line indicates equal contribution (33.3%) among the three leaves.

Previous results showed that mid-canopy leaves (LFT2–LFT4) contribute most of the SPAD signal used for nitrogen inference. We therefore examined whether the relative feature importance among these key leaf positions changes with development. Plant nitrogen concentration (PNC) was used as a representative target, and Random Forest (RF) was used to compute per-sample attributions. For each observation, the absolute contributions of LFT2–LFT4 were normalized so that LFT2 + LFT3 + LFT4 = 100%, and trends were summarized along the GDD axis using nonparametric smoothing with bootstrap confidence bands (Figure 5).

A pronounced redistribution of leaf importance was observed. At early physiological time (~650–1000 °C·d), LFT2 dominated the within-set importance, accounting for roughly half of the LFT2–LFT4 signal, whereas LFT3 contributed ~30% and LFT4 remained lowest (~10–15%). As development progressed, LFT2 importance declined sharply while LFT3 increased, yielding a clear crossover. The LFT2–LFT3 equality point occurred at 1066 °C·d, with a bootstrap 95% confidence interval of 1042–1138 °C·d (Figure 5). Beyond this window, LFT3 became the dominant contributor, stabilizing at approximately ~45–55% of the LFT2–LFT4 importance across mid-to-late GDD.

In parallel, LFT4 showed a gradual late-season rise, increasing from ~10–15% early to ~25–35% in mid/late physiological time. This rise coincided with the sustained weakening of LFT2, such that LFT4 approached—and in parts of late development matched or exceeded—LFT2, although the transition was less sharply localized than the LFT2–LFT3 crossover (as reflected by wider overlap of bootstrap bands). Overall, these RF-based dynamics indicate that the most important leaf within the mid-canopy shifts from LFT2 in early growth to LFT3 after ~1.1 × 10^3^ °C·d, with increasing late-season contribution from LFT4 rather than a fixed ranking throughout the season.

### 2.5. Model Simplification Highlights Two-Leaf Protocols as Practical Alternatives to the Full Set

**Figure 6:**
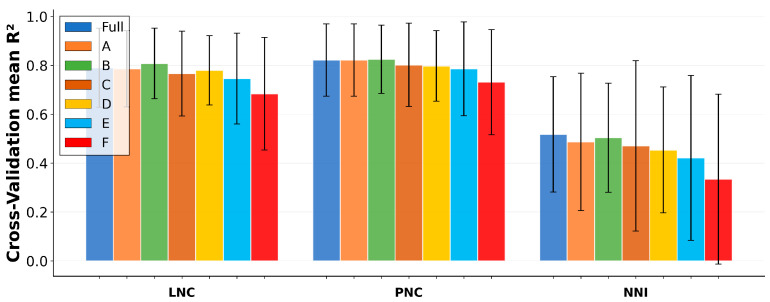
Cross-validated R^2^ for full and simplified SPAD sampling protocols. Bars show mean R^2^ across 10 folds; error bars indicate standard deviation across folds. Protocols include the full feature set (LFT1–LFT5, GDD), three two-leaf protocols (A–C), and three single-leaf protocols (D–F). Targets are LNC, PNC, and NNI.

Model simplification was evaluated using 10-fold GroupKFold cross-validation under a consistent RF configuration, comparing the full protocol (LFT1–LFT5 with GDD) against six reduced-input protocols (Figure 6). Overall, two-leaf protocols preserved most of the predictive signal for concentration targets, whereas NNI was more sensitive to protocol reduction. For LNC, the full protocol achieved R^2^ = 0.790 ± 0.162. Among simplified protocols, the best two-leaf option was Protocol B (LFT2, LFT4, GDD) with R^2^ = 0.807 ± 0.144, slightly exceeding the full protocol while also reducing the SPAD inputs from five leaves to two. For PNC, two-leaf protocols were essentially indistinguishable from the full protocol (full: R^2^ = 0.822 ± 0.148; best two-leaf: R^2^ = 0.825 ± 0.139). For NNI, the full protocol remained the best-performing configuration (R^2^ = 0.518 ± 0.236). Two-leaf protocols approached but did not surpass the full protocol, and single-leaf protocols showed pronounced degradation (LFT4, GDD: 0.335 ± 0.347). These results indicate that NNI retains meaningful dependence on broader canopy information beyond a single leaf position, even when physiological time is included.

## 3. Discussion

### 3.1. Phenological Bias and Physiological Time Alignment

SPAD–nitrogen relationships in rice are known to vary across developmental stages, cultivars, and leaf traits such as specific leaf weight, which introduces a risk of directional error when SPAD is treated as phenology-invariant [10,13,29]. In this study, GDD was used as a pragmatic proxy for developmental progression. The results support the interpretation that incorporating physiological time as an independent predictor changes the error structure of SPAD-based models [9,33]; rather than only altering goodness-of-fit metrics, it reduces systematic residual patterns along the developmental axis. This pattern is consistent with prior reports that SPAD–N relationships drift with phenology and that physiological-time indices can improve comparability of in-season diagnostics across environments [9,10,33].

From a biological perspective, this behavior is consistent with decomposition in which SPAD reflects instantaneous leaf greenness and nitrogen-related status at specific canopy strata, whereas GDD captures time-structured changes in concentration that are associated with growth, dilution, and age-related processes [35]. Such a separation aligns with the conceptual basis of growth-linked nitrogen dilution theory, where concentration dynamics are strongly coupled to development and biomass accumulation [18,19,20]. In contrast, formulations that rely solely on multiplicative coupling between SPAD and GDD can entangle opposing developmental tendencies (e.g., increasing GDD but non-monotonic SPAD trajectories), which may partially explain the poorer generalization observed for purely multiplicative forms.

### 3.2. Target-Dependent Reliance on GDD Versus Canopy SPAD Signals

Feature attribution analyses suggest that the balance between physiological time and canopy greenness is target dependent. Concentration-type targets (LNC and PNC) place substantial weight on GDD, whereas the integrated sufficiency indicator (NNI) is comparatively more dependent on multi-leaf SPAD structure. This pattern is consistent with the definition of NNI as a relative index normalized by a critical nitrogen baseline, which makes it sensitive to canopy-level nitrogen distribution and plant structural status, rather than to instantaneous concentration alone [36,37,38]. Importantly, these attributions quantify how the fitted models weight correlated predictors for prediction and should not be interpreted as evidence of direct physiological causation.

Across targets, the mid-canopy leaves contribute disproportionately to the SPAD signal, with LFT2–LFT4 consistently carrying most of the attribution mass while the youngest (LFT1) and the lowest leaf (LFT5) contribute less. This result is in line with prior evidence that the optimal SPAD measurement position depends on canopy stratification and diagnostic goal [13,29], and it provides a quantitative basis for prioritizing mid-canopy measurements in practical protocols.

### 3.3. Interpreting Leaf-Position Effects Along Development: Shifting Feature Importance Across Canopy Strata

Beyond target dependence, the importance of individual mid-canopy leaves is not fixed over the season [39,40]. The continuous analysis along the GDD axis shows a redistribution of relative importance among LFT2–LFT4 during development, indicating that the most important leaf can change as physiological time advances. This observation is consistent with a physiological explanation in which the canopy stratum is most informative for model-based nitrogen inference shifts with development, reflecting changes in leaf function, nitrogen allocation, and the onset of senescence-related processes [41,42].

In rice, nitrogen remobilization is strongly linked to leaf growth and senescence [24,40]. Because these processes alter within-canopy nitrogen and chlorophyll gradients, the diagnostic value of a given leaf position can vary as the crop transitions from vegetative growth to reproductive development. The observed temporal redistribution of feature importance therefore reinforces the need to represent physiological time explicitly when SPAD measurements are used for nitrogen diagnosis, and it cautions against assuming a single universally optimal leaf throughout the season.

### 3.4. Simplified Measurement Protocols: What Can Be Reduced Without Losing Robustness

A practical objective of this study is to identify simplified, field-deployable protocols that retain acceptable accuracy under grouped validation. The protocol-reduction results show that two-leaf models can approximate the full multi-leaf baseline for concentration targets [43], and in some cases match or marginally exceed the baseline, suggesting redundancy among leaf-position SPAD features once physiological time is included. In contrast, NNI exhibits higher sensitivity to protocol reduction: although two-leaf models remain competitive, the full protocol generally retains the best mean performance, consistent with the broader information requirement of integrated sufficiency diagnostics [13,34].

Single-leaf protocols further reduce measurement burden but show a clearer performance loss, indicating that at least two canopy strata are typically needed to represent nitrogen-related canopy structure in a way that generalizes across environments. Overall, the results support a deployment strategy in which simplified protocols are selected based on the target variable: two-leaf protocols can be used for concentration diagnosis with limited loss of accuracy, whereas NNI diagnosis benefits from retaining richer canopy information.

From an application standpoint, these results suggest that SPAD can be used more reliably for in-season N management when measurements are interpreted on a physiological-time axis rather than assumed to be phenology-invariant. Practically, incorporating GDD alongside SPAD helps reduce stage-dependent drift and improves cross-environment portability, which supports using time-aligned prediction (or time-stratified decision thresholds informed by GDD) for topdressing decisions. Our findings also support target-dependent sampling; for concentration-oriented diagnosis (LNC/PNC), two mid-canopy leaves combined with GDD can provide a field-efficient option with minimal loss of accuracy, whereas for integrated sufficiency diagnosis (NNI), retaining richer canopy information is advisable.

### 3.5. Limitations and Outlook

Several limitations should be noted. First, crossover points and the detailed shape of leaf-importance trajectories along GDD should be interpreted as dataset-specific tendencies rather than universal constants, given known sensitivities of degree-day approaches to threshold choice and temperature-response nonlinearity. Second, attribution-based interpretations (e.g., SHAP) are explanatory rather than causal; importance reflects the model’s use of correlated predictors under the training distribution, not direct physiological causation.

Future work should test the stability of the observed patterns across wider climates, management regimes, and genotype panels, and evaluate alternative physiological-time definitions where appropriate. For NNI, tighter coupling between statistical models and critical nitrogen dilution frameworks may improve robustness, given the structured, stage-dependent nature of critical N relationships in rice. Additionally, extending from leaf-level SPAD to canopy-profile information (e.g., vertical nitrogen distribution estimation) may further improve integrated sufficiency inference.

## 4. Materials and Methods

### 4.1. Study Sites and Field Experiments

Field experiments were conducted at five rice-producing locations in the middle-lower Yangtze River region of China: Changshu (31°32′56″ N, 120°41′55″ E), Yixing (31°17′19″ N, 119°54′42″ E), Jurong (32°01′15″ N, 119°14′53″ E), Jiaxing (30°49′46″ N, 120°43′15″ E), and Yichun (28°16′57″ N, 115°06′29″ E). These sites span humid subtropical rice systems, with a mean annual temperature of approximately 15.1–17.5 °C, annual precipitation of about 1019–1615 mm, and annual sunshine duration of roughly 1711–2116 h. Soils are classified according to the Chinese Soil Taxonomy and can be expressed in WRB/FAO terms as Anthrosols at Changshu, Yixing, Jurong, and Jiaxing (Gleyi-Stagnic or Hapli-Stagnic Anthrosols) and as an Argi-Udic Ferrosol at Yichun. The dataset comprised 20 independent field experiments (hereafter, experiment groups), covering japonica, indica, and intersubspecific hybrid types, and multiple N fertilizer rates, to induce a wide range of crop N status under diverse environments (Table 3). For each experiment, plots were arranged in a randomized complete block design with 3–4 replicates per N treatment; across experiments, N rates ranged from 0 to 300 kg N ha^−1^. Phosphorus and potassium fertilization, irrigation, and pest/weed control followed locally recommended high-yield management, with N rate as the primary experimental factor. All trials followed a transplanting system. To control seedling-age effects, seedlings were transplanted at a standardized three-leaf-one-heart stage (three fully expanded leaves plus one newly emerging central leaf) across experiments.

### 4.2. Sampling Scheme and Measurements of Plant Traits

Sampling was conducted at key rice growth stages (i.e., tillering, stem elongation, panicle initiation, heading, and grain filling). At each sampling event, 3–6 uniformly growing plants were destructively collected from each plot. Plants were separated into leaves, sheaths, and panicles (when present), oven-killed at 105 °C for 30 min, and then dried at 75 °C to constant mass. Dry biomass was recorded, samples were ground, and nitrogen concentration was determined using the Kjeldahl method [44]. Leaf nitrogen concentration (LNC, %) was determined on leaf tissue. Plant nitrogen concentration (PNC, %) was calculated as aboveground total N accumulation divided by aboveground dry mass.

Immediately before each destructive sampling event, SPAD readings were collected on the same plants selected for biomass and nitrogen measurements. SPAD was measured on the 1st to 5th fully expanded leaves from the top (denoted as LFT1–LFT5) using a chlorophyll meter (SPAD-502Plus, Konica Minolta, Inc., Tokyo, Japan). For each leaf, three SPAD values were recorded around the middle region, avoiding the midrib, and averaged [13]. These multi-leaf SPAD features were used as the primary predictors for LNC and PNC estimation.

### 4.3. Growing Degree Days (GDD) and Nitrogen Nutrition Index (NNI) Calculation

Daily minimum and maximum air temperatures were obtained from the China Meteorological Data Service Center (https://data.cma.cn/, accessed on 1 December 2025), using observations from the nearest meteorological station. All trials were conducted under a transplanting system, and seedlings were transplanted at a standardized three-leaf-one-heart stage. Therefore, the reported sowing date refers to nursery seeding. GDD (°C·d) were calculated as cumulative thermal time from sowing to each sampling date:

$$GDD=\sum_{d=1}^{D}max\left(0,\;\frac{T_{max,d}+T_{min,d}}{2}-T_{base}\right)$$

where T_max,d_ and T_min,d_ are daily maximum and minimum temperatures, and T_base_ is the base temperature for rice development (10 °C). If the daily mean temperature was below T_base_, daily GDD was set to 0. The resulting GDD values provided a common physiological time axis for aligning observations across cultivars and environments. Although canopy SPAD measurements were collected after transplanting, accumulating GDD from sowing is widely used in rice phenology and critical N studies and provides a continuous index of physiological age that carries across transplanting without an artificial reset. Using transplanting as the start point would subtract the pre-transplant thermal time and may change alignment if seedling age varies among experiments; we retained the sowing-based definition for consistency and comparability across trials.

NNI was calculated as follows:

$$NNI=PNC/N_{c}$$

where PNC is the plant nitrogen concentration, and N_c_ is the critical plant nitrogen concentration. An NNI close to 1 indicates near-optimal N status; NNI < 1 indicates nitrogen deficiency; and NNI > 1 indicates nitrogen surplus. N_c_ was derived from a published rice critical nitrogen dilution curve (CNDC) [13], expressed as a function of aboveground dry matter (DM):

$$N_{c}=3.44\;{\mathrm{DM}}^{-0.44}$$

where DM is the aboveground dry matter at the sampling time.

### 4.4. Feature Sets, Model Formulations, and Machine Learning Algorithms

#### 4.4.1. GDD-Based Model Formulations

To evaluate how physiological time influences SPAD-based nitrogen inference, we compared four feature formulations constructed from multi-leaf SPAD measurements (LFT1–LFT5) and GDD:

- Model A (SPAD-only): LFT1, LFT2, LFT3, LFT4, LFT5.
- Model B (multiplicative): LFT1×GDD, LFT2×GDD, LFT3×GDD, LFT4×GDD, LFT5×GDD.
- Model C (additive SPAD + GDD): LFT1, LFT2, LFT3, LFT4, LFT5, GDD.
- Model D (additive + interaction): all inputs of model B and model C.

All interaction terms in Model D were included as additional predictors while retaining their corresponding main effects.

#### 4.4.2. Protocol Simplification Features

To examine field-deployable alternatives to measuring five leaf positions, we compared a set of reduced-input protocols that prioritize mid-canopy leaves and their combinations with GDD. The evaluated protocols were as follows:

- Full protocol: LFT1, LFT2, LFT3, LFT4, LFT5, GDD.
- Simplified protocol A: LFT2, LFT3, GDD.
- Simplified protocol B: LFT2, LFT4, GDD.
- Simplified protocol C: LFT3, LFT4, GDD.
- Simplified protocol D: LFT2, GDD.
- Simplified protocol E: LFT3, GDD.
- Simplified protocol F: LFT4, GDD.

These simplified protocols were evaluated for each nitrogen target (LNC, PNC, and NNI) under the same grouped cross-validation framework as the full protocol.

#### 4.4.3. Modeling Methods

To ensure that the conclusions were not dependent on a single algorithmic choice, we trained and evaluated four widely used regression approaches that represent complementary model classes:

Partial Least Squares regression (PLS). A linear latent-variable method suited to correlated predictors; it projects predictors into a small number of components that explain covariance with the response.

Support Vector Regression (SVR). A kernel-based method that fits a nonlinear function with an ε-insensitive loss; an RBF kernel was used to capture nonlinear relationships.

Random Forest regression (RF). An ensemble of decision trees trained on bootstrap-resampled data with randomized feature selection at splits, capturing nonlinearities and interactions.

Extreme Gradient Boosting (XGBoost). A boosted-tree method that sequentially fits trees to correct residuals, with shrinkage and regularization to control complexity.

Because PLS and SVR are scale-sensitive, continuous predictors were standardized within each training fold and the same transformation was applied to the corresponding validation fold. Tree-based methods (RF and XGBoost) were trained on original feature scales.

### 4.5. Model Training, Validation, and Interpretation

#### 4.5.1. Preprocessing and Fixed Model Configurations

All analyses were implemented in Python (v3.12.10). Predictors were processed within each training fold to avoid information leakage. For PLS and SVR, predictors were further standardized by z-scoring within the training fold, and the resulting parameters were applied to the validation fold.

Missing SPAD predictors mainly occurred when the 5th leaf position (LFT5) was not yet present at early stages, or when LFT5 became senescent (yellowed/dried) at late stages and was therefore not measured. For each cross-validation fold, missing predictor values were imputed using the median values estimated from the training split only, and the same imputation parameters were then applied to the corresponding validation split to avoid information leakage. Samples with missing response values were excluded prior to model fitting.

Model hyperparameters were selected using a lightweight grid search conducted on the training data within the cross-validation procedure and were then kept fixed across all targets (LNC, PNC, and NNI) and feature sets to enable fair comparisons. For PLS, the number of latent components was tuned over a small range (2–5) based on feature dimensionality. For SVR, an RBF kernel was used and key hyperparameters (regularization strength C, kernel width γ, and the ε-insensitive margin) were chosen from a coarse grid. RF hyperparameters were selected from a limited grid, with the final model using 500 trees and a fixed random seed with parallel training. For XGBoost, a lightweight grid search was used to select the number of boosting iterations, learning rate, tree depth, subsampling ratios, and L2 regularization; the final configuration used 800 boosting iterations, a learning rate of 0.05, maximum depth of 6, row and feature subsampling ratios of 0.8, and L2 regularization (λ = 1.0), with a fixed random seed.

#### 4.5.2. Group-Aware Cross-Validation and Evaluation Metrics

To prevent leakage across independent field experiments, model performance was assessed using group 10-fold cross-validation (GroupKFold) with splits defined by experiment ID (each independent field experiment). All observations from the same experiment were assigned entirely to either training or validation data within a fold.

Out-of-fold predictions were evaluated using R2, RMSE, and MAE. Metrics were calculated for each fold and summarized as mean ± standard deviation across folds.

#### 4.5.3. Phenological Bias Diagnostics

Phenology-related confounding was diagnosed by testing whether out-of-fold residuals (observed minus predicted) exhibited systematic association with GDD. Pearson correlation and a fitted linear slope between residuals and GDD were computed and contrasted between formulations with and without explicit GDD terms (e.g., SPAD-only vs. SPAD + GDD).

#### 4.5.4. Feature Attribution and Continuous Importance Dynamics Along GDD

For tree-based models (RF and XGBoost), feature contributions were quantified using Shapley additive explanations (SHAP) values. To avoid information leakage, SHAP values were computed in an out-of-fold manner. For each cross-validation fold, models were fitted on the training split and SHAP values were computed for the held-out split; out-of-fold SHAP values were then concatenated across folds for downstream analyses [45,46].

To characterize how the relative importance of key mid-canopy leaves changes continuously with development, analyses focused on LFT2–LFT4. For each observation, absolute SHAP values of LFT2–LFT4 were normalized so that their sum equaled 100%, yielding per-sample relative importance (%). Relative importance was then smoothed as a function of GDD using locally weighted scatterplot smoothing (LOWESS) with a span (fraction) of 0.25 and no robustifying iterations (it = 0). The span was selected to balance noise reduction with preservation of gradual changes in importance across development, and robustifying iterations were not used to avoid over-smoothing transitional patterns. The smoothed curves were evaluated on a common grid of 220 equally spaced GDD points spanning the observed GDD range. Uncertainty bands were obtained via group-level bootstrap resampling (800 replicates); in each replicate, all groups were resampled with replacement (sampling the same number of groups as observed), and the LOWESS curves were re-estimated. Pointwise 95% confidence intervals were derived from the 2.5th and 97.5th percentiles of the bootstrap distribution. Crossover GDD values where the LFT2 and LFT3 curves intersect were computed in each bootstrap replicate as the first sign change on the grid, using linear interpolation between adjacent grid points, and the 95% confidence interval of the crossover was reported from the bootstrap distribution.

## 5. Conclusions

Across grouped multi-environment validation, adding physiological time (GDD) to multi-leaf SPAD models improved the robustness of rice N diagnosis by reducing phenology-driven directional errors in concentration inference. The balance between GDD and SPAD was target dependent: LNC/PNC relied strongly on GDD, whereas NNI depended more on multi-leaf canopy SPAD structure. Leaf-position information was concentrated in mid-canopy strata and shifted along physiological time, supporting simplified, target-specific measurement protocols. From a practical perspective, these results suggest that SPAD can be used more reliably for in-season N management when measurements are interpreted on a physiological-time axis rather than assumed to be stage-invariant, and incorporating GDD alongside SPAD improves cross-environment portability and can reduce the risk of stage-dependent misdiagnosis. In field deployment, two mid-canopy leaves combined with GDD provide a workload-efficient option for diagnosing concentration targets, whereas integrated sufficiency diagnosis (NNI) benefits from retaining richer canopy information.

## Figures and Tables

**Table 1:** Descriptive statistics of key variables.

Variable	Mean	Median	IQR (Q3–Q1)	SD
NNI	0.95	0.96	0.34	0.24
LNC (%)	3.02	2.88	1.07	0.82
PNC (%)	2.02	1.85	1.08	0.76
LFT1 SPAD	39.14	39.04	5.00	3.88
LFT2 SPAD	42.43	42.70	5.25	4.17
LFT3 SPAD	42.33	42.80	5.63	4.54
LFT4 SPAD	41.31	42.17	6.69	6.06
LFT5 SPAD	39.17	40.88	9.26	7.59
Mean SPAD	41.23	41.83	5.32	4.50

NNI = nitrogen nutrition index; LNC = leaf nitrogen concentration; PNC = plant nitrogen concentration; SD = standard deviation; IQR = interquartile range; LFTx = raw (pre-calibration) SPAD for the x-th leaf from the top fully expanded leaf.

**Table 2:** GroupKFold cross-validation performance for four regressors under four feature constructions.

Input Variable	Target Variable	Model	R^2^	RMSE	MAE
SPAD only	LNC	PLS	0.43 ± 0.25	0.57 ± 0.072	0.44 ± 0.073
		SVR	0.47 ± 0.32	0.54 ± 0.110	0.39 ± 0.092
		RF	0.49 ± 0.3	0.53 ± 0.088	0.39 ± 0.086
		XGBoost	0.43 ± 0.32	0.56 ± 0.086	0.42 ± 0.081
	PNC	PLS	0.43 ± 0.29	0.52 ± 0.073	0.41 ± 0.064
		SVR	0.5 ± 0.36	0.47 ± 0.100	0.34 ± 0.093
		RF	0.51 ± 0.33	0.47 ± 0.086	0.34 ± 0.090
		XGBoost	0.47 ± 0.37	0.49 ± 0.094	0.36 ± 0.095
	NNI	PLS	0.31 ± 0.23	0.18 ± 0.025	0.15 ± 0.018
		SVR	0.37 ± 0.4	0.17 ± 0.041	0.13 ± 0.030
		RF	0.41 ± 0.26	0.17 ± 0.027	0.13 ± 0.022
		XGBoost	0.37 ± 0.27	0.17 ± 0.026	0.14 ± 0.021
SPAD×GDD	LNC	PLS	0.24 ± 0.24	0.67 ± 0.095	0.55 ± 0.087
		SVR	0.33 ± 0.40	0.61 ± 0.100	0.47 ± 0.095
		RF	0.22 ± 0.48	0.65 ± 0.140	0.47 ± 0.110
		XGBoost	0.17 ± 0.50	0.67 ± 0.140	0.50 ± 0.120
	PNC	PLS	0.31 ± 0.29	0.58 ± 0.067	0.47 ± 0.059
		SVR	0.41 ± 0.40	0.52 ± 0.100	0.40 ± 0.096
		RF	0.30 ± 0.50	0.56 ± 0.140	0.42 ± 0.120
		XGBoost	0.24 ± 0.51	0.59 ± 0.130	0.44 ± 0.110
	NNI	PLS	0.099 ± 0.18	0.22 ± 0.034	0.18 ± 0.030
		SVR	0.014 ± 0.44	0.22 ± 0.042	0.17 ± 0.034
		RF	0.033 ± 0.31	0.22 ± 0.043	0.18 ± 0.036
		XGBoost	−0.097 ± 0.43	0.23 ± 0.044	0.19 ± 0.036
SPAD + GDD	LNC	PLS	0.71 ± 0.12	0.41 ± 0.066	0.33 ± 0.059
		SVR	0.77 ± 0.11	0.36 ± 0.057	0.28 ± 0.045
		RF	0.79 ± 0.16	0.33 ± 0.091	0.26 ± 0.073
		XGBoost	0.73 ± 0.17	0.38 ± 0.110	0.29 ± 0.074
	PNC	PLS	0.77 ± 0.13	0.33 ± 0.051	0.27 ± 0.043
		SVR	0.81 ± 0.11	0.30 ± 0.046	0.23 ± 0.041
		RF	0.82 ± 0.15	0.27 ± 0.073	0.21 ± 0.058
		XGBoost	0.77 ± 0.15	0.32 ± 0.079	0.25 ± 0.062
	NNI	PLS	0.39 ± 0.23	0.17 ± 0.029	0.14 ± 0.023
		SVR	0.47 ± 0.24	0.16 ± 0.026	0.13 ± 0.020
		RF	0.52 ± 0.23	0.15 ± 0.023	0.12 ± 0.018
		XGBoost	0.51 ± 0.24	0.15 ± 0.024	0.12 ± 0.017
SPAD + GDD + SPAD×GDD	LNC	PLS	0.70 ± 0.16	0.41 ± 0.081	0.33 ± 0.078
		SVR	0.74 ± 0.15	0.38 ± 0.067	0.29 ± 0.054
		RF	0.80 ± 0.16	0.32 ± 0.093	0.25 ± 0.075
		XGBoost	0.74 ± 0.17	0.37 ± 0.110	0.28 ± 0.071
	PNC	PLS	0.76 ± 0.16	0.33 ± 0.065	0.27 ± 0.058
		SVR	0.79 ± 0.11	0.31 ± 0.050	0.24 ± 0.039
		RF	0.82 ± 0.15	0.27 ± 0.072	0.21 ± 0.057
		XGBoost	0.78 ± 0.14	0.31 ± 0.080	0.24 ± 0.055
	NNI	PLS	0.40 ± 0.21	0.17 ± 0.027	0.14 ± 0.021
		SVR	0.42 ± 0.31	0.17 ± 0.030	0.13 ± 0.023
		RF	0.51 ± 0.23	0.15 ± 0.023	0.12 ± 0.018
		XGBoost	0.51 ± 0.22	0.15 ± 0.023	0.12 ± 0.018

Values denote mean ± SD across folds. LNC = leaf nitrogen concentration (%); PNC = plant nitrogen concentration (%); NNI = nitrogen nutrition index (dimensionless); RMSE = root mean squared error; MAE = mean absolute error; PLS = partial least squares regression; SVR = support vector regression; RF = random forest regression; XGBoost = extreme gradient boosting; GDD = growing degree days (°C·d).

**Table 3:** Summary of the 20 field experiments.

No.	Cultivars	Subspecies	Rice Type	Number of Nitrogen Treatment	Experimental Site	Sowing Date
1	NG46_1	Japonica	Conventional	6	Changshu	14 May 2018
2	NG46_2	Japonica	Conventional	6	Changshu	14 May 2019
3	NG46_3	Japonica	Conventional	6	Yixing	16 May 2018
4	NG46_4	Japonica	Conventional	6	Yixing	16 May 2019
5	WYG35	Japonica	Conventional	6	Yixing	12 May 2018
6	NG5055	Japonica	Conventional	7	Yixing	19 May 2019
7	NG3908	Japonica	Conventional	7	Yixing	23 May 2019
8	J67	Japonica	Conventional	3	Jiaxing	13 May 2019
9	XS14	Japonica	Conventional	6	Jiaxing	10 May 2019
10	JH218	Japonica	Conventional	5	Jiaxing	12 May 2019
11	YG13	Japonica	Conventional	5	Jurong	25 May 2019
12	YY15	Intersubspecific hybrid	Hybrid	4	Jiaxing	19 May 2019
13	CY5	Japonica	Hybrid	4	Yichun	25 June 2019
14	CY6	Japonica	Hybrid	5	Changshu	13 May 2019
15	JYZK6	Japonica	Hybrid	2	Jiaxing	15 May 2019
16	HHZ	Indica	Conventional	4	Yichun	25 June 2019
17	MXXZ	Indica	Conventional	4	Yichun	25 June 2019
18	TY398	Indica	Hybrid	4	Yichun	25 June 2019
19	TYXZ	Indica	Hybrid	4	Yichun	25 June 2019
20	TYHZ	Indica	Hybrid	4	Yichun	25 June 2019

Each numbered entry represents an independent field experiment. For cultivar NG46, the suffixes denote four independent experiments (two at Changshu and two at Yixing), rather than distinct cultivars. Sowing date refers to nursery seeding prior to transplanting and is reported as month–day. Seedlings were transplanted at a uniform three-leaf-one-heart stage across experiments. All experiments used a randomized complete block design with 3–4 replicates per nitrogen treatment.

## Data Availability

Data will be made available upon request.

## Abbreviations

Growing Degree Days (GDD), Soil–Plant Analysis Development (SPAD), Leaf Nitrogen Concentration (LNC), Plant Nitrogen Concentration (PNC), Nitrogen Nutrition Index (NNI), Fully expanded Leaves From the Top (LFT), Partial Least Squares (PLS), Support Vector Regression (SVR), Random Forest (RF), Extreme Gradient Boosting (XGBoost), SHapley Additive exPlanations (SHAP), Group K-Fold Cross-Validation (GroupKFold), Locally Weighted Scatterplot Smoothing (LOWESS).
